# Supplementary material for: Mapping the evolution of fertility support policies in China: A content and instrumental analysis
Source: PLoS One. 2025 Oct 9;20(10):e0332137. doi: 10.1371/journal.pone.0332137 (PMC12510515; doi:10.1371/journal.pone.0332137)
Supplement: S1 Appendix — (ZIP) [file pone.0332137.s001.zip › S1 Appendix. 226 original policy documents/99-女职工劳动保护特别规定.doc]

女职工劳动保护特别规定

**发布部门：** [国务院](https://www.pkulaw.com/law/chl?Aggs.IssueDepartment=5)

**发文字号：**中华人民共和国国务院令第619号

**发布日期：**2012.04.28

**实施日期：**2012.04.28

**时效性：** [现行有效](https://www.pkulaw.com/law/chl?Aggs.TimelinessDic=01)

**效力级别：** [行政法规](https://www.pkulaw.com/law/chl?Aggs.EffectivenessDic=XC0201)

**法规类别：** [劳动安全与劳动保护](https://www.pkulaw.com/law/chl?Aggs.Category=08706) [老少妇幼残保护](https://www.pkulaw.com/law/chl?Aggs.Category=01604) [营商环境优化](https://dobusiness.pkulaw.com/)

中华人民共和国国务院令
（第619号）

　　《女职工劳动保护特别规定》已经2012年4月18日国务院第200次常务会议通过，现予公布，自公布之日起施行。

总　理 　温家宝
二○一二年四月二十八日

　　女职工劳动保护特别规定

　　第一条　为了减少和解决女职工在劳动中因生理特点造成的特殊困难，保护女职工健康，制定本规定。

　　第二条　中华人民共和国境内的国家机关、企业、事业单位、社会团体、个体经济组织以及其他社会组织等用人单位及其女职工，适用本规定。

　　第三条　用人单位应当加强女职工劳动保护，采取措施改善女职工劳动安全卫生条件，对女职工进行劳动安全卫生知识培训。

　　第四条　用人单位应当遵守女职工禁忌从事的劳动范围的规定。用人单位应当将本单位属于女职工禁忌从事的劳动范围的岗位书面告知女职工。

　　女职工禁忌从事的劳动范围由本规定附录列示。国务院安全生产监督管理部门会同国务院人力资源社会保障行政部门、国务院卫生行政部门根据经济社会发展情况，对女职工禁忌从事的劳动范围进行调整。

　　第五条　用人单位不得因女职工怀孕、生育、哺乳降低其工资、予以辞退、与其解除劳动或者聘用合同。

　　第六条　女职工在孕期不能适应原劳动的，用人单位应当根据医疗机构的证明，予以减轻劳动量或者安排其他能够适应的劳动。

　　对怀孕7个月以上的女职工，用人单位不得延长劳动时间或者安排夜班劳动，并应当在劳动时间内安排一定的休息时间。

　　怀孕女职工在劳动时间内进行产前检查，所需时间计入劳动时间。

　　第七条　女职工生育享受98天产假，其中产前可以休假15天；难产的，增加产假15天；生育多胞胎的，每多生育1个婴儿，增加产假15天。

　　女职工怀孕未满4个月流产的，享受15天产假；怀孕满4个月流产的，享受42天产假。

　　第八条　女职工产假期间的生育津贴，对已经参加生育保险的，按照用人单位上年度职工月平均工资的标准由生育保险基金支付；对未参加生育保险的，按照女职工产假前工资的标准由用人单位支付。

　　女职工生育或者流产的医疗费用，按照生育保险规定的项目和标准，对已经参加生育保险的，由生育保险基金支付；对未参加生育保险的，由用人单位支付。

　　第九条　对哺乳未满1周岁婴儿的女职工，用人单位不得延长劳动时间或者安排夜班劳动。

　　用人单位应当在每天的劳动时间内为哺乳期女职工安排1小时哺乳时间；女职工生育多胞胎的，每多哺乳1个婴儿每天增加1小时哺乳时间。

　　第十条　女职工比较多的用人单位应当根据女职工的需要，建立女职工卫生室、孕妇休息室、哺乳室等设施，妥善解决女职工在生理卫生、哺乳方面的困难。

　　第十一条　在劳动场所，用人单位应当预防和制止对女职工的性骚扰。

　　第十二条　县级以上人民政府人力资源社会保障行政部门、安全生产监督管理部门按照各自职责负责对用人单位遵守本规定的情况进行监督检查。

　　工会、妇女组织依法对用人单位遵守本规定的情况进行监督。

　　第十三条　用人单位违反本规定第六条第二款、第七条、第九条第一款规定的，由县级以上人民政府人力资源社会保障行政部门责令限期改正，按照受侵害女职工每人1000元以上5000元以下的标准计算，处以罚款。

　　用人单位违反本规定附录第一条、第二条规定的，由县级以上人民政府安全生产监督管理部门责令限期改正，按照受侵害女职工每人1000元以上5000元以下的标准计算，处以罚款。用人单位违反本规定附录第三条、第四条规定的，由县级以上人民政府安全生产监督管理部门责令限期治理，处5万元以上30万元以下的罚款；情节严重的，责令停止有关作业，或者提请有关人民政府按照国务院规定的权限责令关闭。

　　第十四条　用人单位违反本规定，侵害女职工合法权益的，女职工可以依法投诉、举报、申诉，依法向劳动人事争议调解仲裁机构申请调解仲裁，对仲裁裁决不服的，依法向人民法院提起诉讼。

　　第十五条　用人单位违反本规定，侵害女职工合法权益，造成女职工损害的，依法给予赔偿；用人单位及其直接负责的主管人员和其他直接责任人员构成犯罪的，依法追究刑事责任。

　　第十六条　本规定自公布之日起施行。1988年7月21日国务院发布的《[女职工劳动保护规定](https://www.pkulaw.com/chl/133408887a349cdabdfb.html#class=alink)》同时废止。

　　附录：

　　女职工禁忌从事的劳动范围

　　一、女职工禁忌从事的劳动范围：
　　（一）矿山井下作业；
　　（二）[体力劳动强度分级](https://www.pkulaw.com/chl/7652e51bddf12069bdfb.html#class=alink)标准中规定的第四级体力劳动强度的作业；
　　（三）每小时负重6次以上、每次负重超过20公斤的作业，或者间断负重、每次负重超过25公斤的作业。
　　二、女职工在经期禁忌从事的劳动范围：
　　（一）冷水作业分级标准中规定的第二级、第三级、第四级冷水作业；
　　（二）低温作业分级标准中规定的第二级、第三级、第四级低温作业；
　　（三）[体力劳动强度分级](https://www.pkulaw.com/chl/7652e51bddf12069bdfb.html#class=alink)标准中规定的第三级、第四级体力劳动强度的作业；
　　（四）高处作业分级标准中规定的第三级、第四级高处作业。
　　三、女职工在孕期禁忌从事的劳动范围：
　　（一）作业场所空气中铅及其化合物、汞及其化合物、苯、镉、铍、砷、氰化物、氮氧化物、一氧化碳、二硫化碳、氯、己内酰胺、氯丁二烯、氯乙烯、环氧乙烷、苯胺、甲醛等有毒物质浓度超过国家职业卫生标准的作业；
　　（二）从事抗癌药物、己烯雌酚生产，接触麻醉剂气体等的作业；
　　（三）非密封源放射性物质的操作，核事故与放射事故的应急处置；
　　（四）高处作业分级标准中规定的高处作业；
　　（五）冷水作业分级标准中规定的冷水作业；
　　（六）低温作业分级标准中规定的低温作业；
　　（七）高温作业分级标准中规定的第三级、第四级的作业；
　　（八）噪声作业分级标准中规定的第三级、第四级的作业；
　　（九）[体力劳动强度分级](https://www.pkulaw.com/chl/7652e51bddf12069bdfb.html#class=alink)标准中规定的第三级、第四级体力劳动强度的作业；
　　（十）在密闭空间、高压室作业或者潜水作业，伴有强烈振动的作业，或者需要频繁弯腰、攀高、下蹲的作业。
　　四、女职工在哺乳期禁忌从事的劳动范围：
　　（一）孕期禁忌从事的劳动范围的第一项、第三项、第九项；
　　（二）作业场所空气中锰、氟、溴、甲醇、有机磷化合物、有机氯化合物等有毒物质浓度超过国家职业卫生标准的作业。
